# Formation of a Tunnel under the Major Hepatic Vein Mouths during Removal of IVC Tumor Thrombus

**DOI:** 10.1155/2013/129632

**Published:** 2013-09-26

**Authors:** Dmytro Shchukin

**Affiliations:** ^1^Kharkov National Medical University, 4 Lenina Avenue, Kharkov 61022, Ukraine; ^2^V.I. Shapoval Regional Clinical Center of Urology and Nephrology, 195 Moskovskiy Avenue, Kharkov 61037, Ukraine

## Abstract

This study describes a clinical observation of successful implementation of a new surgical maneuver: formation of a cross tunnel under the mouths of the major hepatic veins during removal of a tumor thrombus of the inferior vena cava. This surgical technique helps avoid the usage of   “piggyback” mobilization of the liver and the complications associated with it. However, for objective evaluation of this approach, a further clinical study is required.

## 1. Introduction

Obvious limitations of high tumor thrombus removal with the use of balloon catheters or cardiopulmonary bypass have determined the development of alternative methods making it possible to fully control subhepatic, retrohepatic, and intrapericardial segments of the inferior vena cava (IVC) [1, 2]. The most important aspect of this approach is feasibility of external digital displacement of the thrombus apex below the diaphragm [3, 4]. However, due to the weakness of caval collateral vessel development, the clamping of the IVC above the mouths of the major hepatic veins can cause serious hemodynamic changes. Therefore, the main task of a surgeon in such a situation is to further displace the thrombus downwards and clamp the IVC below the mouths of the major hepatic veins [3, 4]. This maneuver was proposed by Ciancio et al. and now is used by many surgeons worldwide. It helps maintain hepatic blood flow, which accounts for about 25% of blood flow to the inferior vena cava. The main condition for the implementation of this stage of the operation is to carry out the mobilization of the liver using the classical and “piggyback” variants. But we should be aware that due to the problems associated with the venous anatomy, “piggyback” liver mobilization is possible only in 80%–92% of cases [5, 6].

This study presents a clinical observation of removal of a high tumor thrombus of the IVC with the use of a surgical technique, which involves forming a cross tunnel under the mouths of the major hepatic veins without “piggyback” mobilization of the liver.

## 2. Case Presentation

**Figure 1:**
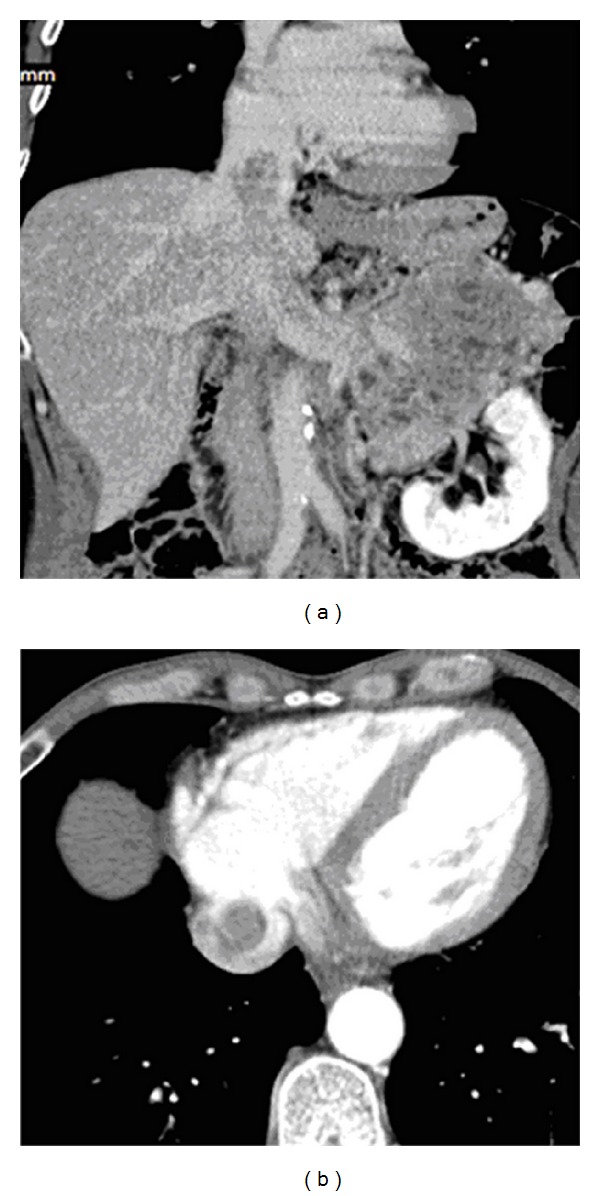
MSCT images of supradiaphragmatic IVC tumor thrombus ((a) frontal reconstruction and (b) axial scan at the level of intrapericardial part of IVC).

A 57-year-old man was admitted to the hospital with complaints of gross hematuria and general weakness. Examination revealed a left kidney tumor, 150 mm × 120 mm in size, extending up to the intrapericardial segment of the inferior vena cava (Figure 1). Distant and regional metastases were not found. Ultrasonography demonstrated thrombus apex flotation, which suggested the absence of adhesions of intraluminal tumor to the vena cava endothelium.

**Figure 2:**
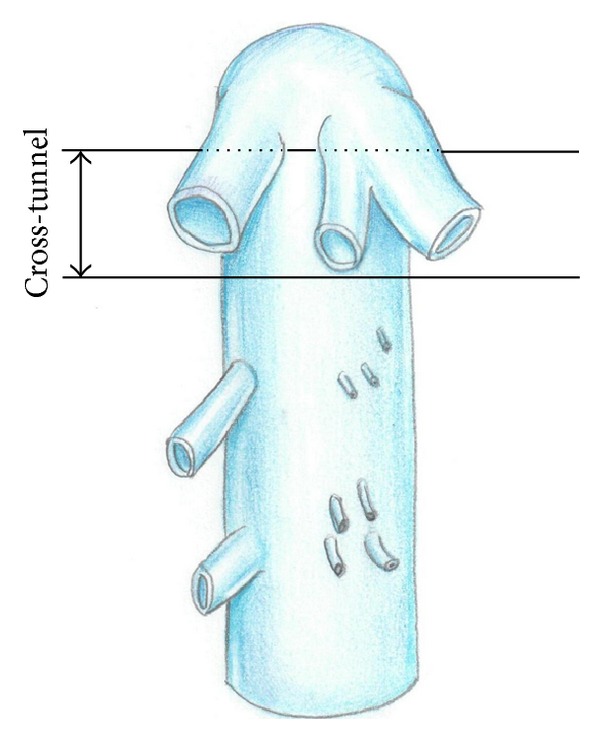
The layout of a cross tunnel below the mouth of the major hepatic veins.

The patient underwent laparotomy through “chevron” incision. The duodenum was mobilized using Kocher's method. The inferior vena cava was exposed in the subrenal and subhepatic segment. The left renal artery was ligated. Falciform, both triangular and coronary, ligaments of the liver were transected. Suprahepatic infradiaphragmatic part of the inferior vena cava with the terminal portions of the major hepatic veins was exposed. The right lobe of the liver was rotated medially. A great number of dorsal hepatic veins were found to drain into the area of retrohepatic inferior vena cava front wall. Hepatic tissue at this level surrounds no more than half the circumference of the IVC. After careful exposure of major hepatic vein mouths, directly below them between the front surface of the IVC and the rear surface of the liver, a tunnel about 1.0 cm wide was formed bluntly and sharply (Figure 2).

**Figure 3:**
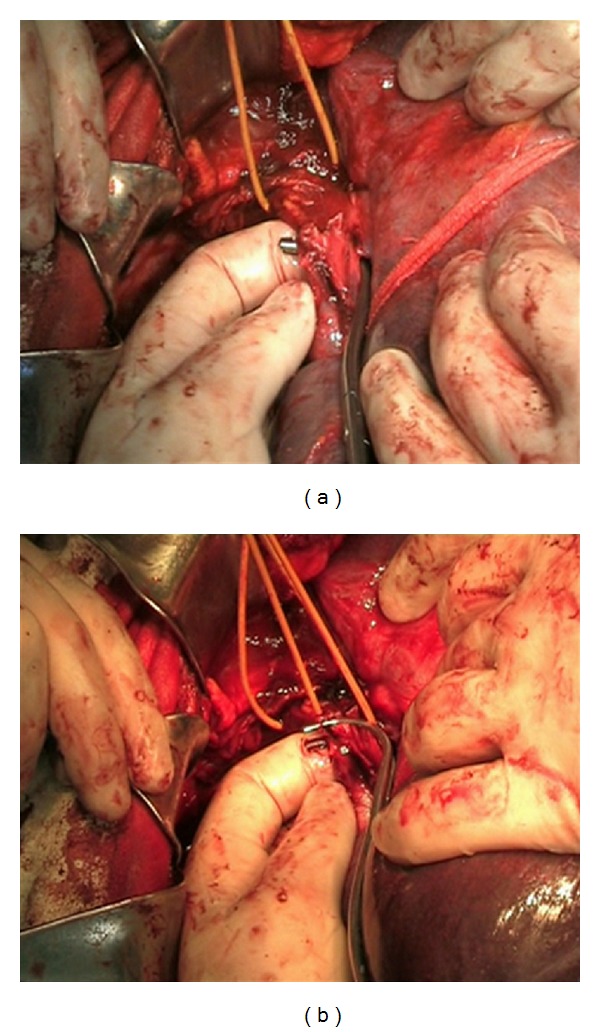
(a) Vascular clamp passed through the cross tunnel below the mouths of the major hepatic veins. (b) Thrombus milked below the mouths of the major hepatic veins. The clamp was placed above the apex of the thrombus.

After T-shaped dissection of the diaphragm in the area of its caval opening, the intrapericardial inferior vena cava was exposed without opening the pericardium. The thrombus was milked manually below the mouths of the major hepatic veins. Through the preformed tunnel under the mouths of the major hepatic veins, a vascular clamp was passed and placed above the top of the thrombus (Figure 3). Isolation of the intraluminal portion of the tumor was finished by clamping the right renal vein and IVC below the thrombus. A slight decrease of blood pressure (to 100/60 mm Hg) was noted. The IVC was dissected longitudinally in its subhepatic segment. The tumor thrombus was removed along with the left kidney. The IVC was covered with twisted 4/0 prolene suture. The wound was drained and stitched. The postoperative period was uneventful. The patient was discharged from the hospital 10 days after the surgery.

## 3. Discussion

**Figure 4:**
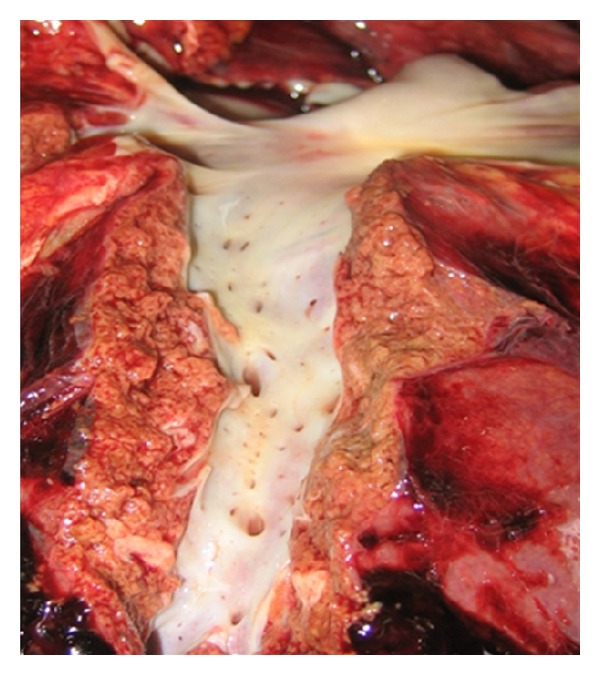
Autopsy observation: in the retrohepatic IVC more than 25 entrances of the dorsal hepatic veins open.

The main hepatic veins and the veins of the caudate lobe of the liver in a number of observations have a very short extrahepatic portion, quite thin walls, and a variable location. In some patients, more than ten veins opening into the retrohepatic IVC were found (Figure 4). Therefore, the use of “piggyback” mobilization of the liver in some cases is associated with serious surgical difficulties and complications.

From our point of view, “piggyback” mobilization of the liver during removal of a high IVC tumor thrombus is not always required. In particular, when the liver covers less than half the circumference of the retrohepatic vena cava. At the same time, in order to bring the thrombus downwards, it is enough to perform mobilization of the liver using the classic version and to release the posterior surface of the vena cava.

Taking into account the geometrical features of the retrohepatic IVC and major hepatic veins, as well as the imaging findings, we have assumed that there is an avascular zone immediately below the mouths of the major hepatic veins, which is about 1.0 cm wide, through which a vascular clamp can be passed without performing the “piggyback” mobilization of the liver. Thus, a surgeon with his hand above the thrombus apex and grasping the vena cava posteriorly and laterally, rather than circularly, can easily displace the thrombus below the mouths of the major hepatic veins. At the same time, the clamp above the top of the thrombus can be passed through the cross tunnel directly under the mouths of the major hepatic veins.

To confirm this hypothesis, we conducted a prior anatomical study of the retrohepatic IVC for the assessment of feasibility and risk level of two options for surgical approaches to this segment of the IVC: “piggyback” mobilization of the liver and creation of a cross tunnel under the mouths of the major hepatic veins (the data is not yet published). “Piggyback” mobilization of the liver was defined as impossible in 11.4% of cases. In 68.6% of cases, it was difficult to perform. With respect to making a tunnel under the mouths of the major hepatic veins, it should be noted that its formation was not possible in 20% of cases. However, in 31.4% of patients, this maneuver was carried out easily. Injury of the liver parenchyma, hepatic veins, or inferior vena cava took place in 14.3% of cases using “piggyback” mobilization of the liver, whereas formation of a tunnel under the mouths of hepatic veins caused the similar problems in 28.6% of cases.

This clinical observation presents a successful result of the use of surgical techniques for creating a cross tunnel under the mouths of the major hepatic veins.

We observed no significant difficulties in performing this maneuver, but one should keep in mind that the tumor thrombus had a small diameter, the liver covered less than half the circumference of the retrohepatic vena cava, and the dorsal hepatic veins penetrated the IVC 1 cm below the mouths of the major hepatic veins.

For more detailed and objective evaluation of this approach, a further clinical investigation is required. There is no doubt that intraoperative ultrasonography of the liver can greatly facilitate the performance and reduce the risk level during creating a tunnel under the mouths of the major hepatic veins.

## 4. Conclusion

This clinical observation has demonstrated the ability to perform a new maneuver during removal of the tumor thrombus of the inferior vena cava by the way of creation of cross tunnel under the mouths of the major hepatic veins. This surgical technique helps avoid the use of “piggyback” mobilization of the liver and the complications associated with it. However, for objective evaluation of this approach, a further clinical study is required.
